# Biopharmaceutics of Topical Ophthalmic Suspensions: Importance of Viscosity and Particle Size in Ocular Absorption of Indomethacin

**DOI:** 10.3390/pharmaceutics13040452

**Published:** 2021-03-26

**Authors:** Elisa Toropainen, Sara J. Fraser-Miller, Dunja Novakovic, Eva M. Del Amo, Kati-Sisko Vellonen, Marika Ruponen, Tapani Viitala, Ossi Korhonen, Seppo Auriola, Laura Hellinen, Mika Reinisalo, Unni Tengvall, Stephanie Choi, Mohammad Absar, Clare Strachan, Arto Urtti

**Affiliations:** 1 School of Pharmacy, University of Eastern Finland, 70211 Kuopio, Finland; elisa.toropainen@uef.fi (E.T.); eva.delamo@uef.fi (E.M.D.A.); kati-sisko.vellonen@uef.fi (K.-S.V.); marika.ruponen@uef.fi (M.R.); ossi.korhonen@uef.fi (O.K.); seppo.auriola@uef.fi (S.A.); laura.hellinen@uef.fi (L.H.); mika.reinisalo@uef.fi (M.R.); unni.tengvall@uef.fi (U.T.); 2 Drug Research Program, Division of Pharmaceutical Chemistry and Technology, University of Helsinki, 00014 Helsinki, Finland; sara.miller@otago.ac.nz (S.J.F.-M.); dunja.novakovic@helsinki.fi (D.N.); tapani.viitala@helsinki.fi (T.V.); clare.strachan@helsinki.fi (C.S.); 3 Food and Drug Administration, Rockville, MD 20993, USA; Stephanie.Choi@fda.hhs.gov (S.C.); abir.absar@astrazeneca.com (M.A.); 4 AstraZeneca Pharmaceuticals, Rockville, MD 20878, USA; 5 Drug Research Program, Division of Pharmaceutical Biosciences, University of Helsinki, 00014 Helsinki, Finland; 6 Laboratory of Biohybrid Technologies, Institute of Chemistry, St. Petersburg State University, 198504 Peterhof, Russia

**Keywords:** ocular absorption, indomethacin, suspension, bioequivalence, dissolution, particle size, viscosity

## Abstract

Eye drops of poorly soluble drugs are frequently formulated as suspensions. Bioavailability of suspended drug depends on the retention and dissolution of drug particles in the tear fluid, but these factors are still poorly understood. We investigated seven ocular indomethacin suspensions (experimental suspensions with two particle sizes and three viscosities, one commercial suspension) in physical and biological tests. The median particle size (d_50_) categories of the experimental suspensions were 0.37–1.33 and 3.12–3.50 µm and their viscosity levels were 1.3, 7.0, and 15 mPa·s. Smaller particle size facilitated ocular absorption of indomethacin to the aqueous humor of albino rabbits. In aqueous humor the AUC values of indomethacin suspensions with different particle sizes, but equal viscosity, differed over a 1.5 to 2.3-fold range. Higher viscosity increased ocular absorption 3.4–4.3-fold for the suspensions with similar particle sizes. Overall, the bioavailability range for the suspensions was about 8-fold. Instillation of larger particles resulted in higher tear fluid AUC values of total indomethacin (suspended and dissolved) as compared to application of smaller particles. Despite these tear fluid AUC values of total indomethacin, instillation of the larger particles resulted in smaller AUC levels of indomethacin in the aqueous humor. This suggests that the small particles yielded higher concentrations of dissolved indomethacin in the tear fluid, thereby leading to improved ocular bioavailability. This new conclusion was supported by ocular pharmacokinetic modeling. Both particle size and viscosity have a significant impact on drug concentrations in the tear fluid and ocular drug bioavailability from topical suspensions. Viscosity and particle size are the key players in the complex interplay of drug retention and dissolution in the tear fluid, thereby defining ocular drug absorption and bioequivalence of ocular suspensions.

## 1. Introduction

**Figure 1:**
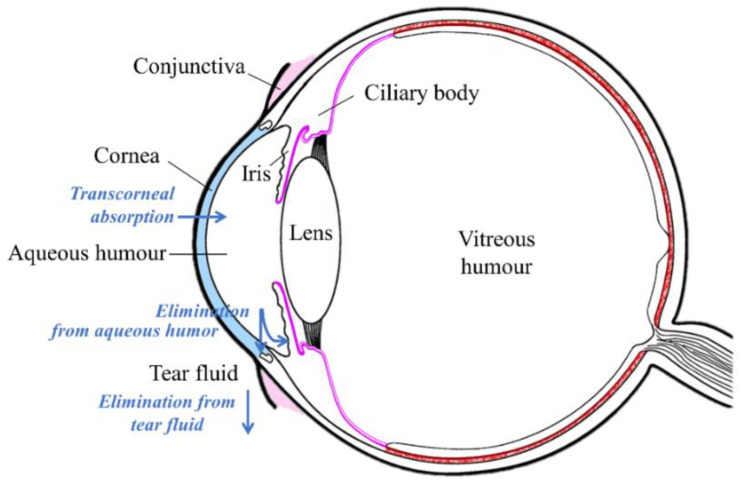
Illustration of topical ocular drug administration and pharmacokinetics.

Topically applied eyedrops are the most commonly used dosage form in ocular drug treatment. They are used in the treatment of anterior segment diseases, such as infections, inflammatory conditions, and glaucoma. Nevertheless, ocular bioavailability of drugs after eyedrop administration to rabbits is limited to less than 5% of the instilled dose, often less than 1% [1]. This is due to several limiting factors: (1) the permeation barrier of the corneal epithelium [2]; (2) rapid drainage of the instilled drug from the ocular surface [3]; and (3) effective transconjunctival drug absorption into the systemic blood circulation [4,5]. In humans, the blinking rate and solution drainage from the ocular surface is also fast and the corneal barrier is tight, indicating low ocular bioavailability [6]. Topical ocular drug delivery is illustrated in Figure 1.

Drug concentrations in ophthalmic eyedrop solutions are usually in the range of 0.1% to 4%, but such drug concentrations in solution are difficult to reach for hydrophobic drugs, and therefore many topical eyedrop formulations are suspensions. Even though ophthalmic suspensions are commonly used, their biopharmaceutical properties are poorly understood. Very few systematic studies involving ocular suspensions have been published, and several studies date from the 1980s and 1990s [7,8,9,10,11,12]. These pivotal reports demonstrate that particle size affects the ocular absorption of dexamethasone [7] and fluorometholone [8] from topically instilled suspensions in rabbits. Increased drug absorption from nanosized particles has also been observed [13,14,15,16,17,18]. These studies suggest that dissolution of suspended particles plays a role in ocular drug absorption.

Biopharmaceutical impact and interplay of formulation factors, such as viscosity and particle size, have not been systematically investigated or modeled. In the eye, these factors may have a significant impact on ocular drug absorption because the typical eye drop volume (30–50 µL) is much larger than the normal volume of weakly buffered lacrimal fluid (7 µL) [19]. Therefore, it is likely that the formulation features (pH, viscosity, excipients) of the eyedrop determine the physical–chemical conditions on the ocular surface and, therefore, significantly influence drug absorption from the topical ophthalmic suspensions.

It is important to build a better understanding of drug delivery from ophthalmic suspensions. Understanding the critical biopharmaceutical factors of ocular topical suspensions would help in the design of optimized formulations for poorly soluble drugs. On the other hand, understanding of the suspensions should foster the regulatory guidance for bioequivalent generic ocular suspensions, thus avoiding expensive and time-consuming pharmacokinetic studies. Aqueous humor sampling from patients undergoing cataract surgery is recommended by the FDA to investigate bioequivalence of corticosteroid suspensions [20].

We investigated seven indomethacin suspensions (one commercial and six experimental formulations) using physical characterization methods and ocular drug absorption studies in vivo in rabbits. Kinetic simulation models were utilized to aid in interpretation of the data.

## 2. Materials and Methods

*Materials*: One commercial indomethacin suspension (Indom^®^ 0.5%) and six test suspensions (INDO1, INDO2, INDO3, INDO4, INDO5, INDO6) with 0.5% of indomethacin were investigated. Indom^®^ 0.5% suspension (Alfa Intes, Lot. 4115 Exp. 08.2017) was purchased from Casoria, Italy. The excipients of Indom^®^ 0.5% include hydroxypropyl methylcellulose (HPMC) 4000, sodium dihydrogen phosphate dihydrate, disodium phosphate dodecahydrate, sodium chloride, edetate disodium, methylparaben, propylparaben, and purified water. Excipient concentrations were analyzed with UPLC (see Supplementary Materials).

Pharmaceutical grade indomethacin from Orion Pharma (Espoo, Finland) was used to prepare the six test suspensions (INDO1-INDO6). HPMC E5 was obtained from Dow Chemicals (Dow Chemicals, Midland, MI, USA), HPMC 4000 was obtained from Sigma-Aldrich (Sigma-Aldrich, St. Louis, MO, USA), while the HPMC K35M (Benecel™) was kindly gifted by Ashland (Ashland, Covington, KY, USA). Sodium dihydrogen phosphate, disodium phosphate, sodium chloride, ethylenediaminetetra acetic acid (EDTA), methylparaben, and propylparaben were USP grade (where applicable) and obtained from Sigma-Aldrich (Sigma-Aldrich, St. Louis, MO, USA). Deionized water was Milli-Q grade.

*Preparation of the suspensions*: Test formulations (INDO1-INDO6) differed in terms of viscosity and particle size, according to Table 1. Viscosity was varied by using different grades of HPMC, in order to obtain low (≈1.3 mPa·s, HPMC E) (IND01 and IND04), medium (≈7 mPa·s, HPMC 4000) (IND02 and IND05), and high (15 mPa·s, HPMC K35M) (IND03 and IND06) viscosity formulations. In addition, test suspensions had either small (IND01-IND03) or large (IND04-IND06) particle sizes that were obtained by wet-milling. Table 2 contains a summary of ingredients and their respective concentrations for each of the test suspensions (50 mL).

*Wet milling*: Wet milling was carried out using a Pulverisette 7 (Fritsch, Idar-Oberstein, Germany) mill equipped with 45 mL zirconium oxide bowls and 70 g of 1 or 5 mm diameter pearls. Micronized indomethacin (2 g) was milled in stabilizer solution (10 mL) with 1 mm diameter pearls at 700 rpm for 6 × 3 min cycles with 15 min cooling breaks to produce small particles approximately 600 nm in diameter. Larger particles were produced by milling with 5 mm diameter pearls for one cycle at 1000 rpm. HPMC 4000 (0.5 wt.%) was used as a stabilizer. The drug and stabilizer solution were both added into the milling bowl before milling was initiated. The remaining formulation components (as indicated in Table 2), along with 1.5 g of the milled suspension, were added to pre-dissolved HPMC solution of predefined grade. The formulations with the same particle size (INDO1-INDO3 and IND04-IND06) were prepared separately using the same milling conditions.

*pH and buffer capacity*: The pH was measured using a Fieldlab pH meter with a Schott Blueline 16pH probe. The system was calibrated with pH 4 and 7 buffer solutions. The buffer capacity of the commercial suspensions was measured (in combination with pH) to determine the concentration of buffer in the commercial suspensions. Buffer capacity (*β*) is related to the change in number of moles of an acid or base (*dn*) and the associated change in pH (*dpH*) for 1 L equivalent of solution: *β* = *dn/dpH*.

The pH of 2 mL of the commercial suspensions was measured before and after addition of 500 µL of sodium hydroxide (0.104 mol/L) to the Indom suspension.

*Osmolality*: The osmolality of the ocular suspensions was measured using an auto-osmometer osmostat OM-6020 (Daiichi Kagaku, Kyoto, Japan). The osmometer was calibrated with Milli-Q water and 300 and 1000 mOsm/kg standard solutions.

*Viscosity*: The viscosity of the Indom suspension was measured using an AR2000 rheometer (TA instruments, New Castle, DE, USA), which was controlled using Rheology Advantage software. A cup and rotor setup were used, consisting of the standard cup with a DIN rotor (TA instruments, New Castle, DE, USA). Viscosity of INDO1-6 was estimated based on HPMC grade specifications.

*X-ray powder diffraction*: X-ray powder diffraction (XRPD) for solid-state form confirmation was carried out using a D8 discover XRD system (Bruker Optik, Ettlingen, Germany). The primary side configuration consisted of a Cu-tube, WL = 1.54 Å; motorized slit (variable); and a 2.5° axial soller. The secondary side configuration included a 3° anti-scatter slit, 2.5° axial soller, 0.02 mm Ni-filter, and a LYNXEYE detector (Bruker, Karlsruhe, Germany) in 1D mode, fully open (192 slits). For the bulk powders the standard sample holder was used. The parameters for the bulk powders consisted of an angular range of 3° to 41°, step size of 0.0161°, and time/step of 0.36 s to give a total measurement time of 15 min. The slit was varied between 12 and 22 mm based on the size of a smooth sample surface. The particles from the suspensions were measured using a zero-background sample holder. The variable slit was set to 4 mm.

*Particle size distribution*: Laser light diffraction for particle sizing was carried out with a Mastersizer 2000 setup including the Hydro 2000s attachment (Malvern Instruments Ltd., Worchestershire, UK). The background was collected using Milli-Q water. The solutions were measured diluted in Milli-Q water (~200 mL) to concentrations that gave the appropriate response levels. The results were calculated based on the default refractive index (*n* = 1.52). Five or 10 measurements were collected and averaged. A pump/stirrer speed of 2975 rpm was used to stir the sample during measurements.

*Indomethacin absorption experiments*: New Zealand albino rabbits (weight 2.5–3.0 kg) were used in the experiments. The rabbits were handled in accordance with the statement of the Animals in Research Committee of ARVO (Association for Research in Vision and Ophthalmology, Rockville, Maryland, MD, USA). All animal experiments were approved by the national Animal Experiment Board of Finland (Eläinkoelautakunta, ELLA; license ESAVI/8893/04.10.07/2014). The animals were kept in conventional housing units involving set temperatures, humidity and a 12/12 h light/dark cycle. The animals lived under normal diet, and they were housed freely on floors in the animal rooms. Indomethacin formulations (INDO1, INDO2, INDO3, INDO4, INDO5, INDO6, Indom) were tested in vivo in rabbits for kinetics in the tear fluid, cornea, and aqueous humor.

*Tear fluid sample collection*: The ophthalmic suspensions were administered onto the upper cornea-scleral limbus of the rabbit eye (25 µL/eye; *n* = 8–12; indomethacin concentration 0.5%). The tear fluid samples of 1 µL were withdrawn from each eye with disposable microcapillaries (Microcaps, Drummond Scientific, Broomall, PA, USA) at time points 1, 3, 5, 10, 20, 30, 60, and 120 min post-instillation, and the samples were immediately cooled on ice following the storage at −80 °C until further analyses.

*Aqueous humor and cornea sample collection*: The indomethacin suspensions were administered onto the upper cornea-scleral limbus of the rabbit eye (25 µL/eye; *n* = 5 eyes). The animals were sacrificed at designated times (15, 30, 60, 90, 120 and 240 min) after instillation by injecting a lethal dose of pentobarbital (Mebunat vet 60 mg/mL; Orion Pharma) into the marginal ear-vein. The eyes were enucleated, aqueous humor was withdrawn from anterior chamber and corneal tissue was dissected and weighed. Corneal samples were diluted 1:10 with 0.9% sodium chloride and homogenized using Ultra-Turrax^®^. The aqueous humor and corneal samples were frozen in liquid nitrogen and stored at −80 °C prior to the analyses.

*Quantification of indomethacin in biological samples*: Tear fluid samples were thawed on ice for 10 min following 1 min of spinning at 13,000 rpm (4 °C). The samples were diluted with 50% acetonitrile containing internal standard (indomethacin-d4) at 20 ng/mL concentration. Standard curve dilutions (0.5–500 ng/mL; three parallels) were prepared using indomethacin stock solution (1 mg/mL; 50% acetonitrile) with the internal standard. Quality controls were prepared in diluted blank tear fluid samples.

The thawed aqueous humor and cornea samples were diluted 1:5 in 0.9% sodium chloride solution. Diluted samples of 150 µL and internal standard (15 ng/mL) of 10 µL were pipetted into glass vials for drug extraction. The samples were vortexed, and 1 mL of methyl tertiary butyl ether (MTBE) was added to each sample. The samples were shaken vigorously for 10 min, and phases were allowed to separate for 15 min at room temperature. Organic phase containing the extracted indomethacin was transferred to new glass vials and evaporated in a vacuum centrifuge. The dried residue was solubilized in 50 µL of 30% acetonitrile. Standard curves (0.02–500 ng/mL) were prepared in 0.9% sodium chloride, and MTBE extraction was performed as described above.

Quantitative analyses were performed using LC-MS/MS, involving an Agilent 1290 series liquid chromatograph and an Agilent 6495 triple-quadrupole mass spectrometer (Agilent Technologies, Inc., Santa Clara, CA, USA) with electrospray ionization. Separation was performed with a Poroshell 120 SB-C18 column (2.1 × 50 mm, 2.7 μm, Agilent, Santa Clara, CA, USA) and Poroshell 120 SB-C18 guard column (2.1 × 5 mm, 2.7 μm, Agilent). The column was maintained at 50 °C. A binary mobile phase with gradient elution was used (A: 0.1% formic acid in mQ-H_2_O; B: 100% methanol). The gradient was performed as follows: 40% of B mobile phase was increased to 100% of B in 5 min, kept constant for 0.5 min, then 100% of B was decreased to 40% of B in 0.1 min and kept constant for 1.4 min. The total run time was 7 min, the flow rate was 0.3 mL/min, and the injection volume was 2 µL. The MS/MS parameters are presented in Table 3.

*Pharmacokinetic calculations and simulations*: Pharmacokinetic parameters of indomethacin in tear fluid, cornea, and aqueous humor were calculated using non-compartmental data analyses (Phoenix software, version 6.3, Pharsight Inc., St. Louis, MO, USA). The impact of dissolution rate and precorneal retention of indomethacin suspensions on ocular absorption was simulated with STELLA Professional software (v. 10, ISEE Systems, Lebanon, NH, USA). Hypothetical dissolution rates in sink conditions (0 to 0.08 min^−1^) were used in the simulations at time = 0, and thereafter the in vivo dissolution rate was dependent on indomethacin solubility and its dissolved concentration in the lacrimal fluid (for the model, see Figure S6). The dissolution rate was determined with equation: A × K (S-C)/C, where A is the remaining undissolved indomethacin, K is the first-order dissolution rate constant in the sink conditions, S is the water solubility of indomethacin, and C is the simulated concentration of indomethacin in the tear fluid. Loss of free drug from the tear fluid was based on known clearance factors for small molecules [1,4,21], whereas the retention of the suspended particles in the tear fluid was varied using first-order rates of elimination in the range of 0.05 to 0.3 min^−1^ (corresponding to half-lives of 2.3–14 min). Typical small molecule values for corneal permeation, distribution volume in anterior chamber, and elimination from the anterior chamber were used for indomethacin (Table S2).

## 3. Results

### 3.1. Physical and Chemical Characterization of the Suspensions

Particle size, viscosity, pH, and osmolality of one test suspension (INDO5) were adjusted to be similar to the commercial product Indom^®^ (Table 4). The buffer capacity of Indom^®^ was approximately 0.020, which corresponds to buffer component concentrations of 78.2 mM of sodium dihydrogen phosphate and 9.8 mM of disodium phosphate. The concentrations of EDTA (500 µg/mL), methylparaben (205 µg/mL), and propylparaben (260 µg/mL) in Indom were measured by ultra-performance liquid chromatography (UPLC) (see Supplementary Materials).

Particle sizing with laser light diffraction (LLD) shows that different milling conditions resulted in two distinct groups of suspensions with smaller particles (INDO1-INDO3) with median diameter (d_50_) of 0.37–1.33 µm and larger particles (INDO4-INDO6) with d_50_ of 3.12–3.50 µm (Table 4; Figure S3). The commercial suspension (Indom^®^) had a d_50_ of 5.48 µm. Scanning electron microscopy (SEM) images of the suspension particles are shown in Figures S1 and S2. The ranges of particle sizes observed visually in the SEM images were largely similar to those measured with LLD. However, LLD indicated presence of some larger particles that were not observed with SEM. This can be attributed to two factors; firstly, the number of particles inspected with SEM was much smaller than that of LLD, making sub-sampling more of a problem, and secondly LLD reports distribution by volume skewing distribution towards larger particle sizes.

The viscosity of the Indom^®^ suspension was 7 mPa·s. The test suspensions with medium viscosity (INDO2, INDO5) were designed to have similar viscosity to the Indom^®^ suspension. The low viscosity formulations (INDO1, INDO4) had a calculated viscosity of 1.3 mPa·s, and high viscosity formulations (INDO3, INDO6) had a calculated viscosity of 15 mPa·s (Table 4).

XRPD results confirmed that raw indomethacin powder and Indom^®^ suspension had polymorphic form γ (Figure S4). During method development, it was also confirmed that milling did not induce solid state changes, and thus the test suspensions also contained the γ form.

### 3.2. Indomethacin Absorption into Rabbit Eyes

**Figure 2:**
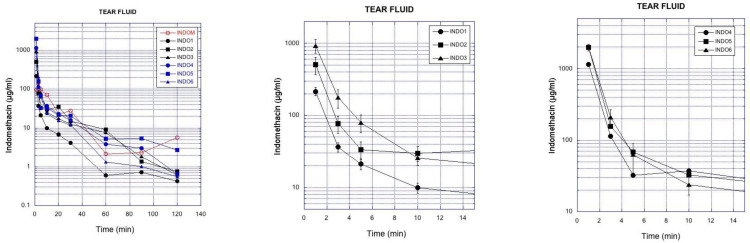
**Left**. Mean concentration of indomethacin in the rabbit tear fluid after instillation of the suspensions (Indom^®^, INDO1-INDO6). **Middle**. Indomethacin concentrations (mean ± SEM) in the tear fluid after instillation of the small particles as low (INDO1), medium (INDO2), and high viscosity (INDO3) suspensions. **Right**. Indomethacin concentrations (mean ± SEM) in the tear fluid after instillation of the large particles as low (INDO4), medium (INDO5), and high viscosity (INDO6) suspensions.

Indomethacin concentrations were monitored in the tear fluid after topical instillation of the ophthalmic suspensions to the rabbit eyes. It is evident that the particle size and viscosity have an impact on the time course of indomethacin concentrations in the tear fluid (Figure 2, left). Increased viscosity in the suspension prolongs the drug retention in the tear fluid, whereas the smaller particle size decreases indomethacin retention in the tear fluid (Figure 2, middle, right).

The data were further analyzed to calculate the AUC values in the tear fluid, based on the mean indomethacin concentrations over time (Table 5). AUC was increased 3.5–4.0-fold with increasing suspension viscosity at similar particle size. Similar fold increase in AUC values at increasing viscosity was seen for small particles and large particles. The concentrations at 1 min (C_1min_) increased 2.4–4.0 times with increasing eyedrop viscosity, but at similar particle size, in the lacrimal fluid. Indomethacin concentrations in the tear fluid (AUC, C_1min_) were smaller after instillation of small particles (INDO1-3) than after application of larger particles (INDO4-6) at similar viscosities.

**Figure 3:**
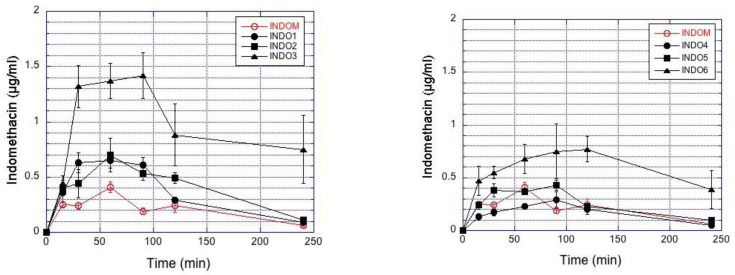
Indomethacin concentrations in the rabbit aqueous humor after topical administration of the suspensions. The results represent the mean ± SEM values in the small (**Left**) and large particle (**Right**) suspensions. The commercial indomethacin suspension (Indom^®^) is included in both figures for reference.

Indomethacin concentrations in rabbit aqueous humor are presented in Figure 3. Particle size and viscosity have influence on the indomethacin concentrations. Increased suspension viscosity (INDO3 > INDO2 > INDO1; INDO6 > INDO5 > INDO4) at similar particle size category increases drug permeation to the aqueous humor for both small and large particles (Figure 3). Indomethacin absorption to the aqueous humor (AUC) after instillation of small particles is higher than that after larger particles with similar viscosity (Table 6). Increasing viscosity improved ocular bioavailability by 3–4 times for both small and large particles (Table 6). Topical application of IND05 and Indom^®^ resulted in similar drug delivery to the aqueous humor (Figure 3, Table 6). Similar trends as in aqueous humor were also seen in the corneal drug levels (Figure S5).

**Figure 4:**
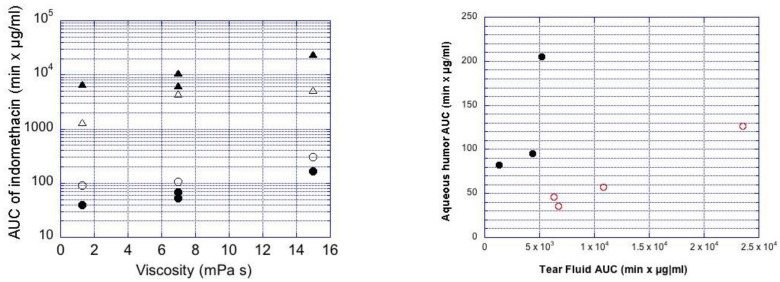
**Left**: Ocular indomethacin pharmacokinetics in the rabbits. AUC values for mean indomethacin concentrations in the aqueous humor (circles) and tear fluid (triangles) are shown. The clear symbols describe the data from small (mean size 0.4–1.3 µm) suspension particles, and the filled symbols are for the large particles (mean size 3.1–3.5 µm) at three different viscosity levels (*x*-axis). **Right**: Plot of AUC in aqueous humor vs. AUC in tear fluid. The filled symbols are for the larger particles (mean size 3.1–3.5 µm) and clear symbols for the smaller particles (mean size 0.4–1.3 µm). Indom is included as medium viscosity formulation with large particles in both figures.

Figure 4 plots the impact of viscosity and particle size on the AUC values in the rabbit aqueous humor. The AUC values increased with decreasing particle size in the aqueous humor, while increasing viscosity increased the AUC values in the aqueous humor. Overall, the range of ocular bioavailability (AUC) is about 8-fold for AUC, and C_max_ shows a 4-fold range (Figure 4).

**Figure 5:**
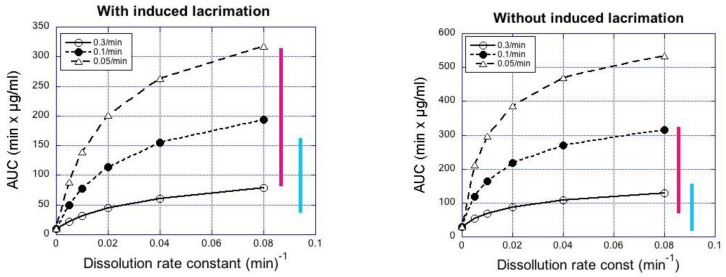
Simulated AUC_0–4h_ values in rabbit aqueous humor as a function of dissolution rate constant at sink conditions. The bars represent the range of experimental AUC_0–4h_ values in rabbits after instillation of large (blue) and small (red) suspension particles.

Compared to the larger particles, the smaller particle sizes delivered higher indomethacin concentrations in the cornea (Figure S5) and aqueous humor (Figure 3), even though the AUC values of indomethacin in the lacrimal fluid were lower for the small particles than for the large particles (Figure 5). Smaller particle size results in increased indomethacin delivery to the aqueous humor when normalized to the AUC values in the tear fluid (Figure 4).

### 3.3. Pharmacokinetic Simulations

Increasing particle retention in the tear fluid (elimination rates: 0.05–0.3 min^−1^ in tear fluid) increased the simulated AUC in the aqueous humor about 4-fold, whereas increasing the rate of dissolution at sink conditions (from 0.01 to 0.08 min^−1^) increased AUC in aqueous humor about 2-fold (Figure 5). The simulated and observed AUC values from Table 6 are in the same range (Figure 5). In the case of simulations with induced lacrimation factor, the AUC levels in aqueous humor after instillation of small particle suspension were in the range of the simulated AUC values at high dissolution rates (≈0.08 min^−1^), whereas the AUC values of large particle suspensions were a better match with the simulated values for slow dissolution. Without induced lacrimation, the simulated AUC values increased to about 1.5-fold higher levels (Figure 5).

## 4. Discussion

The results of this study show that the viscosity of ophthalmic suspensions has a clear impact on ocular indomethacin absorption. Increasing viscosity from 1.3 to 15 mPa resulted in about 4-fold increase in ocular absorption (i.e., AUC in aqueous humor). Thus, viscosity should be an important consideration when ophthalmic suspensions are formulated. Previous studies showed that higher viscosity increases ocular drug absorption from eye drop solutions [22,23] and budesonide suspension [9]. On the other hand, it is known that increased viscosity increases the thickness of unstirred water layer around drug particles [24], but this factor should decrease dissolution rate and reduce drug absorption. Thus, thickening of the unstirred water layer around suspension particles is not important in vivo, and the impact of viscosity on drug retention on the ocular surface is the dominant factor. It should be noted that rheological flow properties have an impact on ocular drug absorption. This is relevant if polymers with non-Newtonian rheology and pseudoplastic spreading characteristics on the ocular surface are used (e.g., caroboxymethylcellulose, hydoxypropylcellulose) [25]. Apparently, higher eyedrop viscosity is able to retain the suspension particles and solution in the tear fluid, as shown in the tear fluid data of indomethacin concentrations (Table 5). It is important to note that capillary samples of tear fluid include both dissolved and undissolved indomethacin.

A smaller particle size in a suspension results in increased surface to volume ratio and faster drug dissolution. In the case of indomethacin, faster dissolution from smaller particles leads to higher ocular drug absorption; the suspension with small particle size (0.4–1.3 µm) showed approximately two times higher drug delivery to the aqueous humor as compared to large particle (3.1–3.5 µm) suspensions. Interestingly, the suspensions with small indomethacin particles were actually removed faster from the tear fluid than the larger particles, as the AUC values in the tear fluid decreased with smaller particle size (Figure 2). Despite their faster lacrimal elimination and lower AUC in tear fluid, the small particle suspensions resulted in improved drug absorption into the eye (Figure 4). An identical relationship between AUC values in tear fluid and in aqueous humor should be seen if the fraction of the dissolved drug in the tear fluid is identical in both cases. Compared to larger particles, a higher proportion of indomethacin dissolves in the tear fluid after application of small particles, thereby leading to improved ocular drug absorption. Thus, retention of the suspension in the tear fluid does not correlate linearly with drug absorption (Figure 5).

Pharmacokinetic simulations were performed to probe the impact of particle dissolution in the ocular indomethacin absorption. Since exact parameter values for indomethacin were not available, we used parameter values that are expected for indomethacin (non-productive clearance from lacrimal fluid with and without induced lacrimation, clearance from lacrimal fluid to the cornea, clearance from aqueous humor) (Figure S6, Table S2). Simulations of indomethacin AUC in the aqueous humor at various retention times and dissolution rates of suspended particles resulted in realistic results that matched the experimental observations, especially when induced lacrimation was included in the model (Figure 4). The simulations reveal some interesting aspects. Firstly, increased drug dissolution rate yields higher ocular bioavailability, but the increase is not linear, suggesting that the sink conditions may not prevail in the lacrimal fluid. Secondly, increased retention of the particles on the ocular surface results in higher AUC in the aqueous humor, but this increase is also non-linear (about 4-fold increase in AUC, whereas the elimination rate constants vary over a 6-fold range). Previous simulations also suggest non-linear behavior of ophthalmic dexamethasone suspensions [26]. We do not know the absolute bioavailability of indomethacin in the aqueous humor because intracameral clearance of indomethacin is not known. However, it is likely that intracameral clearance of indomethacin falls within recently reported range of values (6.4 to 32 µL/min) for intracamerally injected small molecules [27]. With these values we can estimate that ocular bioavailability of indomethacin suspensions with smaller and larger particles is 0.4–1.3% and 0.2–0.7%, respectively.

Our simulations suggest that the concentrations of dissolved indomethacin in the lacrimal fluid at the dissolution rates of 0.1–0.8 min^−1^ are in the range of 3 to 20 and 10 to 23 µg/mL at particle removal rates of 0.05 and 0.3 min^−1^, respectively. Since water solubility of indomethacin at neutral pH is 43 µg/mL, we conclude that dissolution of suspended particles is affected by the dissolved drug in the tear fluid and the sink conditions may not prevail in vivo. Earlier publications suggest that only a fraction of fluorometholone particles dissolve after topical administration in the rabbit eyes [8,10,28]. We can reach this conclusion because the AUC in aqueous humor increased only ≈2–4-fold with suspensions as compared to the fluorometholone solution, even though the drug dose in the suspensions (25 µg) was 67 times higher than in the solution (0.375 µg). Therefore, in vitro dissolution tests overestimate the rate of suspension particle dissolution, and more realistic dissolution methods mimicking drug clearance from the tear fluid might provide more accurate estimation of drug dissolution on the ocular surface.

Bioequivalence of ocular suspensions is not self-evident, even if the suspensions have similar compositions. Particle size distribution may vary due to the suspension processing, and this causes changes in drug dissolution and ocular drug absorption. Here we show that the particle size indeed has influence on drug absorption from the suspensions with equal viscosity (Figure 3). Likewise, differences in viscosity caused changes in indomethacin absorption from suspensions with similar particle sizes (Figure 3). The acceptable range for dissolution rate and viscosity depends on the sensitivity of ocular drug absorption to these parameters. Typically, the AUC values of generic products should be within 80% to 125% of the originator values [29,30]. The changes in particle size and viscosity of the suspensions in this study resulted in greater changes in the AUC values, but the simulations in Figure 4 give some hints about the sensitivity of AUC to the dissolution rate in sink conditions. For example, if we consider the medium viscosity formulation and dissolution rate of 0.04 min^−1^, it seems that dissolution rates of 0.03–0.06 min^−1^ yield mean AUC values within the bioequivalence limits. This theoretical consideration, however, does not take into account important inter-subject variability factors [20]. Clinical variability is also affected by the sedimentation rate of the suspensions. The patients should shake the suspension bottle before eye drop instillation, but the time between shaking, and instillation may vary as well as the sedimentation rate. In this study, the suspensions were instilled immediately after shaking to minimize the impact of sedimentation.

## 5. Conclusions

Biopharmaceutical properties of topical ocular suspensions are still poorly understood. Herein, we demonstrate that particle size and viscosity of indomethacin suspensions affect ocular bioavailability over about an 8-fold range. Increased viscosity increased ocular indomethacin absorption 2.5–3.5-fold for suspensions with similar particle sizes, while smaller particle size increased absorption from equiviscous suspensions 1.6–2.3-fold. Remarkably, total indomethacin concentrations in the tear fluid were disconnected from the ocular bioavailability, as suspensions with larger particles had higher AUC values in the tear fluid than the small particle suspension. These data and simulations suggest that only a fraction of the suspended particles dissolve in tear fluid, and dissolution has a significant impact on ocular drug bioavailability. The published data and pharmacokinetic insights will be useful in the development of original and generic ophthalmic suspensions.

## Figures and Tables

**Table 1:** Preparation conditions for indomethacin formulations.

Sample	Particle Size	Milling Parameters *	Viscosity	Calculated Viscosity (mPa·s)
INDO1	Small	Six cycles at 700 rpm with 1 mm pearls	Low	≈1.3 (HPMC E5)
INDO2	Small	Six cycles at 700 rpm with 1 mm pearls	Medium	≈7 (HPMC 4000)
INDO3	Small	Six cycles at 700 rpm with 1 mm pearls	High	≈15 (HPMC K35M)
INDO4	Large	One cycle at 1000 rpm with 5 mm pearls	Low	≈1.3 (HPMC E5)
INDO5	Large	One cycle at 1000 rpm with 5 mm pearls	Medium	≈7 (HPMC 4000)
INDO6	Large	One cycle at 1000 rpm with 5 mm pearls	High	≈15 (HPMC K35M)

* each test formulation with small (IND01-IND03) or large (IND04-IND06) particle size was prepared separately using the same milling conditions.

**Table 2:** Indomethacin test formulations.

Formulation Component	Role	Concentration
Indomethacin (pre-milled to a certain particle size)	Active pharmaceutical ingredient	0.5 wt.%
HPMC	Wetting and viscosity-increasing agent	0.3 wt.%
Sodium dihydrogen phosphate, disodium phosphate	Buffering agents	78.16 and 9.84 mM
Sodium chloride	Tonicity adjusting agent	35 mM
EDTA	Chelating agent	0.05%
Methylparaben	Preservative	0.0205%
Propylparaben	Preservative	0.026%
Deionized water	Aqueous vehicle	ad 50 mL

**Table 3:** MS/MS parameters of indomethacin analyses. Positive ion mode was used in elestrospray ionization (ESI).

Compound	ESI	Precursor Ion	Product Ion 1	CE (eV)	Product Ion 2	CE (eV)	Internal Standard
Indomethacin	+	358.01	138.9	17	110.9	49	Indomethacin-d4
Indomethacin-d4	+	364.01	143	16			/

**Table 4:** Physical and chemical characteristics of the suspensions.

Sample	Particle Size						Calculated Viscosity (mPa·s)	Osmolality (mOsm/kg)	pH
	Laser Light Diffraction					SEM			
	d_10_ (µm)	d_20_ (µm)	d_50_ (µm)	d_80_ (µm)	d_90_ (µm)	Visual Range (µm)			
INDO1	0.19	0.22	0.43	2.48	5.90	0.1–2	≈1.3 (HPMC E5)	241	5.80
INDO2	0.29	0.45	1.33	7.30	14.00	0.1–3	≈7 (HPMC 4000)	239	5.90
INDO3	0.18	0.22	0.37	3.28	12.81	0.1–4	≈15 (HPMC K35M)	239	5.84
INDO4	0.69	1.22	3.23	5.56	7.21	0.4–4	≈1.3 (HPMC E5)	241	5.82
INDO5	0.74	1.50	3.50	6.03	7.40	0.4–3	≈7 (HPMC 4000)	242	5.89
INDO6	0.80	1.32	3.12	6.40	9.42	0.4–4	≈15 (HPMC K35M)	236	5.91
Indom^®^	0.90	2.75	5.48	9.06	11.40	0.5–5 (bulk), 20–50 (few)	≈7 (measured)	232	5.90

**Table 5:** Pharmacokinetic analysis of the indomethacin concentrations in tear fluid. AUC is the area under the curve until infinity.

Formulation	AUC (min × µg/mL)	CV%	C_1min_ (µg/mL)	CV%
Indom^®^	6312	25	216	33
INDO1	1299	15	63	19
INDO2	4379	17	238	20
INDO3	5197	17	299	20
INDO4	6718	20	400	24
INDO5	10,849	19	445	24
INDO6	23,517	11	1480	13

**Table 6:** Pharmacokinetic parameters of topically applied indomethacin in the rabbit aqueous humor. AUC is the area under the curve until infinity.

Formulation	AUC_0–4h_ (min × µg/mL)	AUC (min × µg/mL)	C_max_ (µg/mL)	T_max_ (min)	Terminal Half-Life (min)
Indom^®^	45.9	52.1	0.012	78	402
INDO1	82.0	89.7	0.008	59	636
INDO2	94.9	104.7	0.009	63	654
INDO3	204.9	301.6	0.032	127	13,826
INDO4	35.5	39.2	0.010	59	256
INDO5	57.5	67.8	0.015	76	422
INDO6	126.3	166.2	0.024	107	744

## Data Availability

Data is contained within the article and Supplementary Materials. The additional data presented in this study are available on request from the authors.

## Supplementary Materials

The following are available online at https://www.mdpi.com/article/10.3390/pharmaceutics13040452/s1, Figure S1: SEM images of the INDO1-INDO3 test suspensions at different magnifications, Figure S2: SEM images of the INDO4-INDO6 suspensions at different magnifications, Figure S3: Particle size distributions of indomethacin suspensions, Figure S4: Calculated diffractograms for α and γ forms of indomethacin compared with the measured diffractograms for solid indomethacin and Indom^®^ solid from suspension, Figure S5: Indomethacin concentrations in the rabbit cornea after topical administration of the suspensions, Figure S6: The simulation model structure, Table S1: The gradient parameters of the mobile phase used for UPLC of the commercial suspensions, Table S2: Pharmacokinetic simulation parameters.
