# Supplementary material for: Biopharmaceutics of Topical Ophthalmic Suspensions: Importance of Viscosity and Particle Size in Ocular Absorption of Indomethacin
Source: Pharmaceutics. 2021 Mar 26;13(4):452. doi: 10.3390/pharmaceutics13040452 (PMC8067094; doi:10.3390/pharmaceutics13040452)
Supplement: Supplementary file 1 [file pharmaceutics-13-00452-s001.pdf]

pharmaceutics

Supplementary Materials: Biopharmaceutics of Topical Ophthalmic Suspensions: Importance of Viscosity and Particle Size in Ocular Absorption of Indomethacin

Elisa Toropainen, Sara J. Fraser-Miller, Dunja Novakovic, Eva del Amo, Kati-Sisko Vellonen, Marika Ruponen, Tapani Viitala, Ossi Korhonen, Seppo Auriola, Laura Hellinen, Mika Reinisalo, Unni Tengvall, Stephanie Choi, Mohammad Absar, Clare Strachan [2] and Arto Urtti

Determination of excipients concentrations in Indom

UPLC was carried out on a Acquity UPLC system (Waters, USA) with an Acclaim Trinity P1 HPLC column (Thermoscientific, USA) with a particle size of 3 µm and dimensions of 2.1 x 50 mm. A flow rate of 0.5 mL/min was used with a column temperature of 30 °C and an injection volume of 5 µL. The mobile phase consisted of two components which were adjusted as a gradient during the measurements. The mobile phases were (A) 50 mM KH_2PO_4 (pH 3.5) and (B) acetonitrile. The gradient table for the measurements is given in Table S1. The detection wavelengths used were 210 nm for EDTA and 255 nm for the parabens. The retention times observed were 3.18 min for EDTA, 4.10 min for methylparaben and 5.00 min for propyl paraben. Samples were repeated using a different dilution factor in 10/90 acetonitrile/phosphate buffer (pH 3.5) and filtered using 0.2µm PTFE filter.

Table S1. The gradient parameters of the mobile phase used for UPLC of the commercial suspensions.

Time	%A	%B
0.00	95.0	5.0
3.00	95.0	5.0
6.00	40.0	60.0
6.01	95.0	5.0
8.00	95.0	5.0

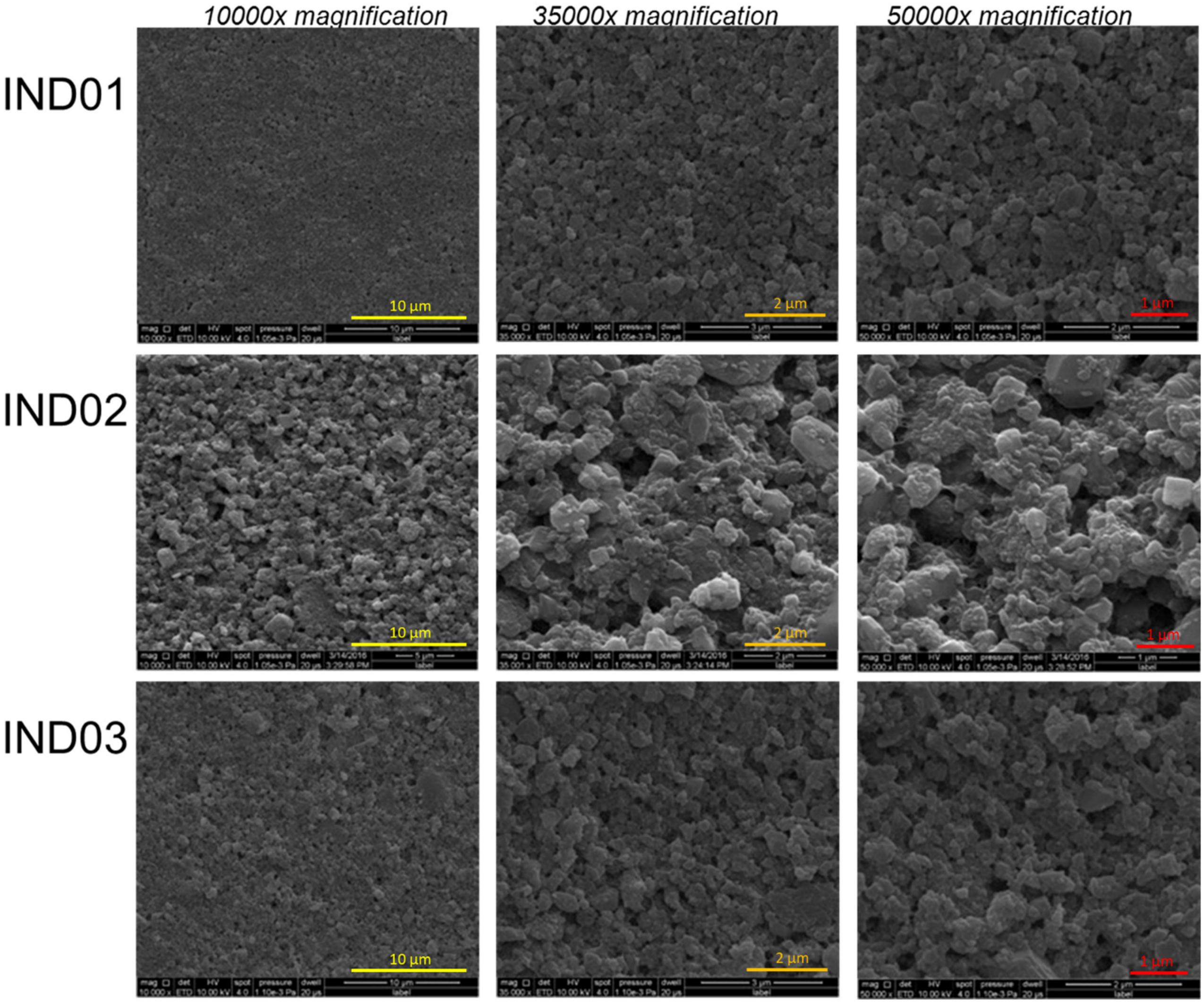

Figure S1. SEM images of the INDO1-INDO3 test suspensions at different magnifications. Three test formulations with small particle size (IND01, IND02 and IND03) were prepared separately using the same milling conditions. Test formulations differed in terms of viscosity, which was achieved by using different grades of HPMC. The yellow denotes a 10 µm scale, orange a 2 µm scale and red a 1 µm scale.

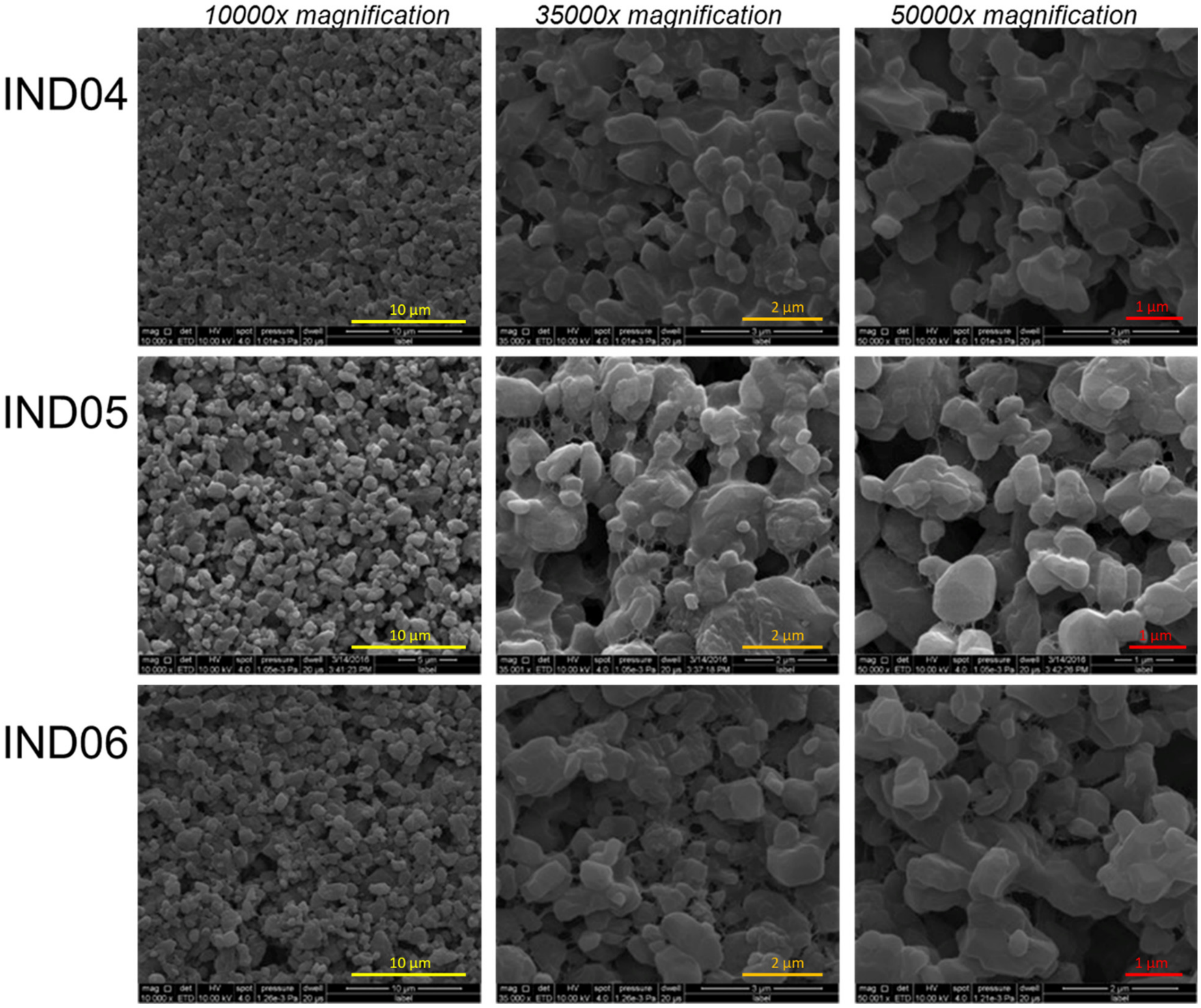

Figure S2. SEM images of the INDO4-INDO6 suspensions at different magnifications. Three test formulations with medium particle size (IND04, IND05 and IND06) were prepared separately using the same milling conditions. Test formulations differed in terms of viscosity, which was achieved by using different grades of HPMC. The yellow denotes a 10 µm scale, orange a 2 µm scale and red a 1 µm scale.

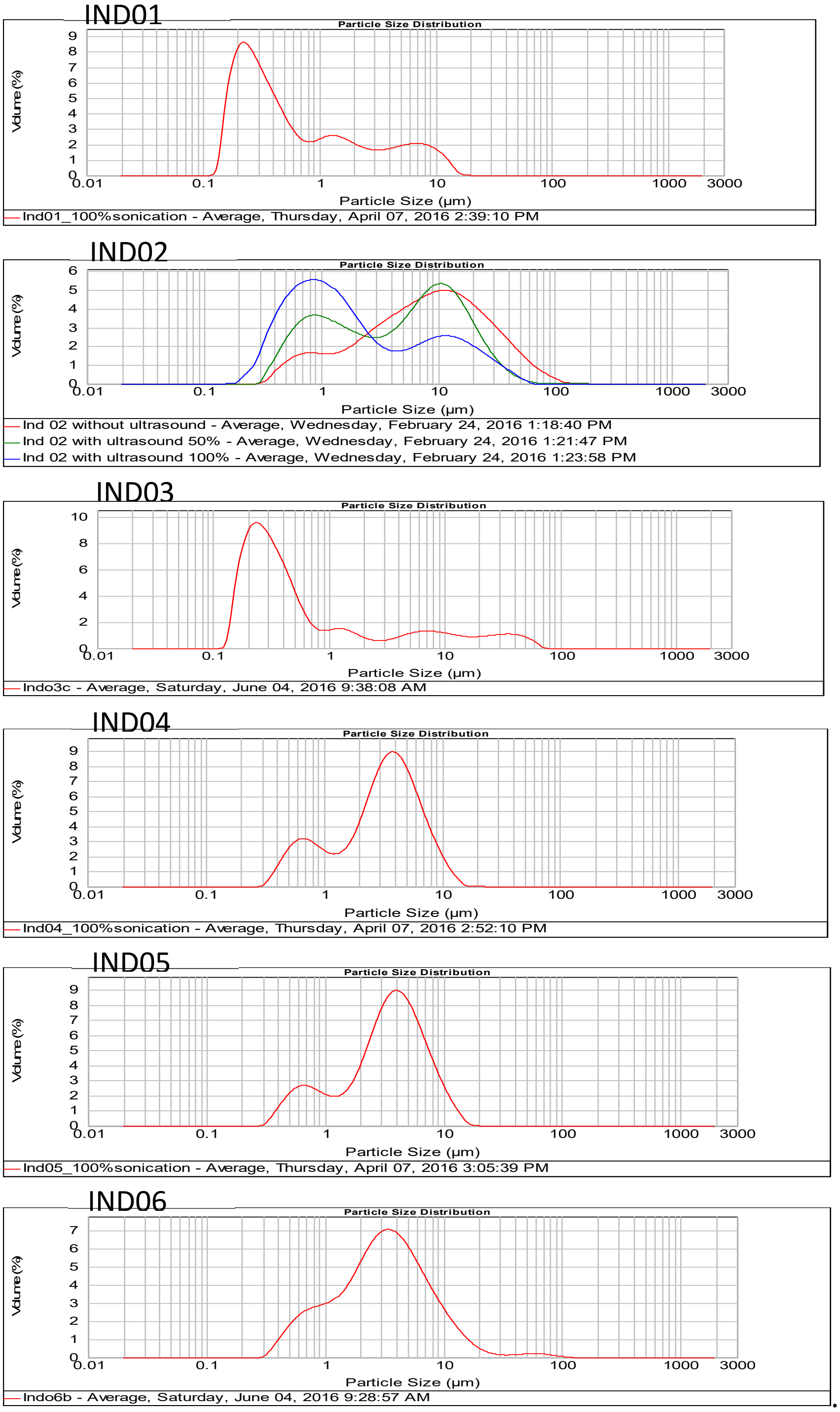

Figure S3. Particle size distributions of indomethacin suspensions. Measurements of all samples except for IND02 only involved 100% ultrasound during the measurement. IND02 sample was measured three times: without sonication, with 50% sonication and with 100% sonication.

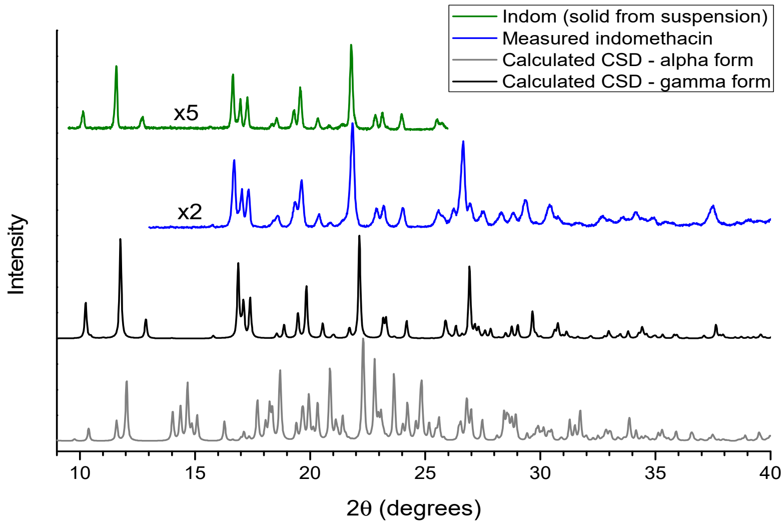

Figure S4. Calculated diffractograms for α and γ forms of indomethacin compared with the measured diffractograms for solid indomethacin and Indom® solid from suspension.

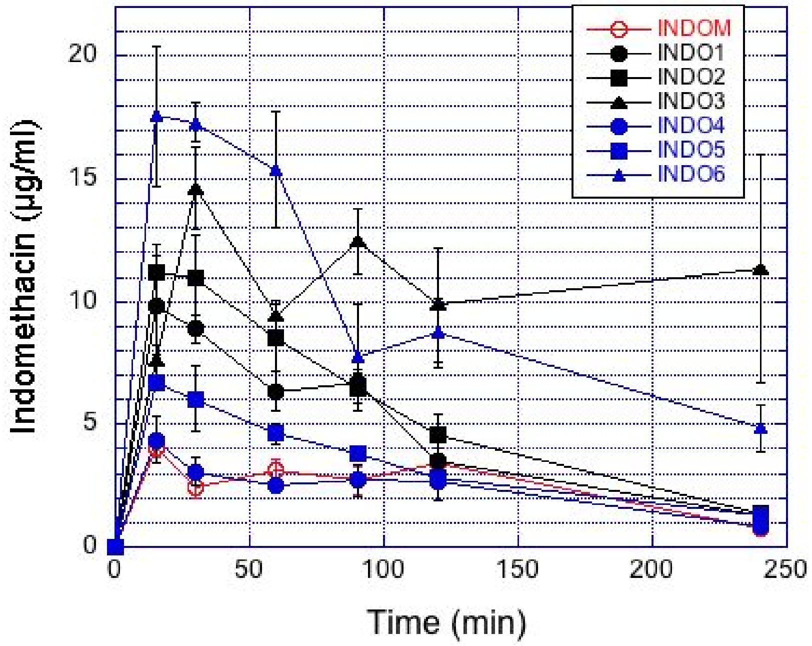

Figure S5. Indomethacin concentrations in the rabbit cornea after topical administration of the suspensions. The results are mean ± SEM values.

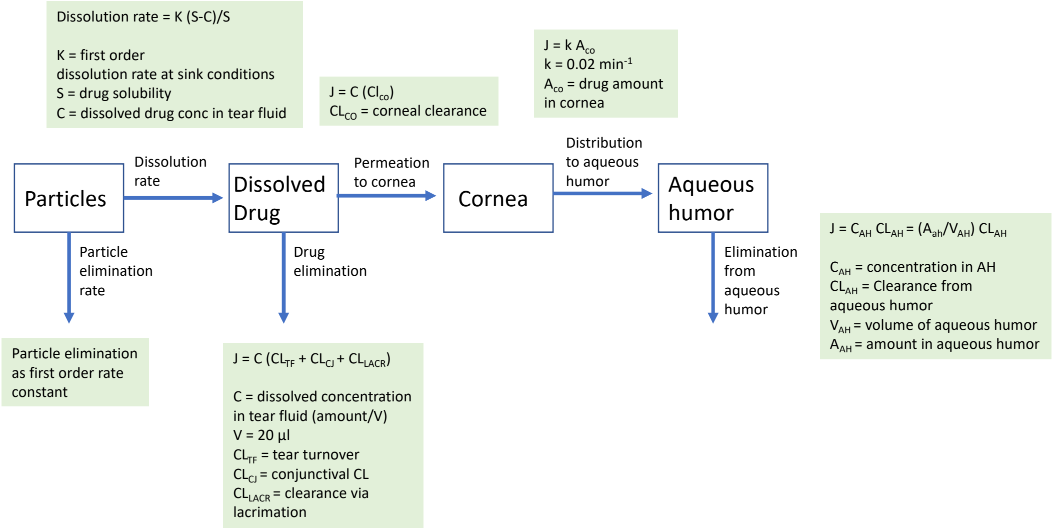

Figure S6. The simulation model structure.

Table S2. Pharmacokinetic simulation parameters.

**Parameter**	**Value**	**Source**
Dissolution rate constant in sink conditions	0-0.8 min^{-1}	Simulated range of values
Water solubility of indomethacin	43 µg/mL	[1]
Clearance of dissolved drug from tear fluid to conjunctiva	7 µL/min	Estimated based on [2]
Clearance of dissolved drug from tear fluid to cornea	1.9 µL/min	Estimated based on [2]
Normal tear turnover	0.5 µL/min	[3]
Induced lacrimation	20 µL/min	[4]
Elimination rate constant of the suspended particles	0.05–0.3 min^{-1}	Simulated range of values
Volume of eye drop and tear fluid	20 µL	Experimental value from this study
Rate constant for drug distribution from cornea to aqueous humor	0.02 min^{-1}	Estimated based on [5]
Volume of distribution in anterior chamber	750 µL	Estimated based on [6]
Indomethacin clearance from aqueous humor	7.5 µL/min	Estimated based on [6]

References

1. Inada, A.; Oshima, T.; Takahashi, H.; Baba, Y.; Enhancement of water-solubility of indomethacin by complexation with protein hydrolysate. *Int. J. Pharm.* **2013**, *453*, 587-593.
2. Ramsay, E.; del Amo, E.; Toropainen, E.; Tengvall-Unadike, U.; Ranta, V.P.; Urtti, A.; Ruponen, M.; Corneal and conjunctival drug permeability: systematic comparison and pharmacokinetic impact in the eye. *Eur. J. Pharm. Sci.* **2018,** *119*: 83-89.
3. Maurice, D.M.; Mishima, S.; Ocular pharmacokinetics. In *Handbook of Experimental Pharmacology, vol 69, Pharmacology of the Eye*. Ed. M.L. Sears, Springer-Verlag, Berlin -Heidelberg, 1984, pp. 16-119.
4. Lee, V.H.L.; Robinson, J.R.; Mechanistic and quantitative evaluation of precorneal pilocarpine disposition in albino rabbits. *J. Pharm. Sci.* **1979**, *68*, 673-684.
5. Makoid, M.C.; Robinson, J.R.; Pharmacokinetics of topically applied pilocarpine in albino rabbit eye. *J. Pharm. Sci.* **1979**; *68*, 435-443.
6. Fayaaz, A.; Ranta, V.; Toropainen, E.; Vellonen, K.S.; D'Amico Ricci, G.; Reinisalo, M.; Heikkinen, E.M.; Gardner, E.; Urtti, A.; Jamei, M.; del Amo, E.M.; Ocular intracameral pharmacokinetics for a cocktail of timolol, betaxolol and atenolol in rabbits. *Mol. Pharmaceut.* **2020**, *17*, 588-594.
